# Membrane composition is a functional determinant of NIR-activable liposomes in orthotopic head and neck cancer

**DOI:** 10.1515/nanoph-2021-0191

**Published:** 2021-07-06

**Authors:** Mina Guirguis, Chanda Bhandari, Junjie Li, Menitte Eroy, Sushant Prajapati, Ryan Margolis, Navadeep Shrivastava, Kenneth Hoyt, Tayyaba Hasan, Girgis Obaid

**Affiliations:** Department of Bioengineering, University of Texas at Dallas, Richardson 75080, Texas, USA.; Department of Bioengineering, University of Texas at Dallas, Richardson 75080, Texas, USA.; Department of Bioengineering, University of Texas at Dallas, Richardson 75080, Texas, USA.; Department of Bioengineering, University of Texas at Dallas, Richardson 75080, Texas, USA.; Department of Bioengineering, University of Texas at Dallas, Richardson 75080, Texas, USA.; Department of Bioengineering, University of Texas at Dallas, Richardson 75080, Texas, USA.; Department of Bioengineering, University of Texas at Dallas, Richardson 75080, Texas, USA.; Department of Bioengineering, University of Texas at Dallas, Richardson 75080, Texas, USA.; Wellman Center for Photomedicine, Massachusetts General Hospital and Harvard Medical School, Boston 02114, Massachusetts, USA; and Division of Health Sciences and Technology, Harvard University and Massachusetts Institute of Technology, Cambridge 02139, Massachusetts, USA.; Department of Bioengineering, University of Texas at Dallas, Richardson 75080, Texas, USA

**Keywords:** head and neck cancer, nanomedicine, near infrared, photodynamic therapy, tumor delivery

## Abstract

Near-infrared (NIR)-activable liposomes containing photosensitizer (PS)-lipid conjugates are emerging as tunable, high-payload, and tumor-selective platforms for photodynamic therapy (PDT)-based theranostics. To date, the impact that the membrane composition of a NIR-activable liposome (the chemical nature and subsequent conformation of PS-lipid conjugates) has on their *in vitro* and *in vivo* functionality has not been fully investigated. While their chemical nature is critical, the resultant physical conformation dictates their interactions with the immediate biological environments. Here, we evaluate NIR-activable liposomes containing lipid conjugates of the clinically-used PSs benzoporphyrin derivative (BPD; hydrophobic, membrane-inserting conformation) or IRDye 700DX (hydrophilic, membrane-protruding conformation) and demonstrate that membrane composition is critical for their function as tumor-selective PDT-based platforms. The PS-lipid conformations were primarily dictated by the varying solubilities of the two PSs and assisted by their lipid conjugation sites. Conformation was further validated by photophysical analysis and computational predictions of PS membrane partitioning (topological polar surface area [tPSA], calculated octanol/water partition [cLogP], and apparent biomembrane permeability coefficient [P_app_]). Results show that the membrane-protruding lipo-IRDye700DX exhibits 5-fold more efficient photodynamic generation of reactive molecular species (RMS), 12-fold expedited phototriggered burst release of entrap-ped agents, and 15-fold brighter fluorescence intensity as compared to the membrane-inserting lipo-BPD-PC (phosphatidylcholine conjugate). Although the membrane-inserting lipo-BPD-PC exhibits less efficient photo-dynamic generation of RMS, it allows for more sustained phototriggered release, 10-fold greater FaDu cancer cell phototoxicity, and 7.16-fold higher tumor-selective delivery in orthotopic mouse FaDu head and neck tumors. These critical insights pave the path for the rational design of emerging NIR-activable liposomes, whereby functional consequences of membrane composition can be tailored toward a specific therapeutic purpose.

## 1 Introduction

Photonanomedicines (PNMs) are light-activable nanoscale drug delivery systems that facilitate photodynamic therapy (PDT). PDT destroys disease tissue through photochemistry, a light-initiated reaction between a light activable molecule – the photosensitizer (PS) – and oftentimes molecular oxygen [1, 2]. Common lipid-based PNM platforms that are frequently used for PDT-based regimens include liposomes, liposome bilayer coated nanoconstructs, and monolayer lipid-coated nanoconstructs. Visudyne^™^, a near-infrared (NIR)-activable liposome containing the PS benzoporphyrin derivative (BPD), was the first PNM to gain Food and Drug Administration approval in 2000 for PDT of Age-related Macular Degeneration [3]. In recent years, NIR-activable PNMs, especially those based on nanometric liposomal formulations, have seen an exponential rise in use as platform technologies that combine a PS for PDT and phototriggered release of secondary therapeutics. Such secondary, and at times tertiary, therapeutics include small molecule inhibitors [4], targeted biologics [5, 6], chemotherapeutics [7–12], and immunotherapeutic agents [13–16]. Salient features that are unique to NIR activation of liposomes include(1) rigorous control over the dose deposition of photodynamic reactive molecular species (RMS) and of entrapped secondary therapeutics, in addition to (2) spatiotemporal control over the induction of therapeutic combination regimens [1, 2].

In recent years, PS-lipid conjugates have emerged as versatile liposome membrane anchors with remarkable optical, photochemical, and phototherapeutic properties, in addition to providing unique stability for PSs within the liposomal membranes [1, 11, 12, 17]. This stability is critical for keeping the PS molecule strongly associated with the liposome during *in vivo* circulation [11], for facilitating true molecular specificity when targeting tumor-associated receptors [1, 18], and for nanoscale precision in targeting various organelles [19–21]. Molecular specificity for receptor-targeted PDT can only truly be achieved when PS molecules are stably anchored by lipids into the bilayer. We have previously demonstrated that BPD nonspecifically and rapidly leaks out of liposomes into nontarget cells [18]. Conjugation of BPD to 16:0 lyso phosphatidylcholine (PC) and 20:0 lyso PC entirely prevents this nonspecific and premature leakage from liposomal membranes [18]. In the case of secondary and tertiary entrapped agents, this degree of stability within a liposome is also critical for the molecular specificity of co-delivery. Even if these agents are released prematurely within the tumor interstitium, it is conceivable that a molecularly targeted liposome containing a stable lipid-conjugated PS will sensitize the target cells to the secondary agents nearby. However, this is specific to the secondary or tertiary agents, their stability within liposomes, their mechanism of action, and the mechanisms of their synergy with PDT [1].

Significant strides have been made in the therapeutic, diagnostic, and theranostic applications of NIR-activable constructs that contain PS-lipid conjugates. Recent noteworthy examples include porphysomes comprised entirely of self-assembled porphyrin-phospholipid conjugates and liposomes doped with porphyrin-phospholipid conjugates entrapping chemotherapy developed by Zheng, Lovell and colleagues [11, 12, 17]. The membrane composition (the chemical nature and resultant physical conformation) of NIR-activable liposomes has only been studied in the context of their impact on the optical properties of the PS and on biophysical self-assembly. Such compositions that lead to membrane-protruding or membrane-inserting conformations include chromophore molecules conjugated to the terminal end of phospholipid acyl chains [22], sn-1 or sn-2 hydroxyl groups of lysophospholipids [17, 18, 21, 23], phosphate headgroups [24, 25], hydroxyl groups of cholesterol [21], and to terminal hydrophilic functional groups of 1,2-distearoyl-*sn*-glycero-3-phosphoethanolamine-polyethylene glycol (DSPE-PEG) [21, 26, 27]. In our prior studies, we reported the first synthesis and characterization of a panel of lipid-based NIR-activable PNMs containing BPD conjugates to 16:0 lyso PC, 20:0 lyso PC, cholesterol, and DSPE-mPEG_2000_-NH_2_ [21]. These constructs demonstrated a high degree of tunability in their optical and photochemical activity [21]. We have also shown that 16:0 lyso PC and 20:0 lyso PC lipid conjugation of BPD resulted in an exclusively lysosomal localization within cancer cells, thereby allowing multi-organelle targeted PDT regimens when used in combination with unmodified BPD liposomes that target the endoplasmic reticulum and mitochondria [19–21]. This strategy has demonstrated superior outcomes in two-dimensional and three-dimensional (3D) cancer models when using a single-wavelength 690 nm irradiation protocol [19–21].

To date, studies have mostly dealt with PS-lipid conjugates as passive spectators in the liposomes whose functions are to facilitate PDT, phototriggered agent release, or a combination of both. Considering the significance and clinical relevance of emerging NIR-activable liposomes, this study explores the *in vitro* and *in vivo* functional impact that modulating the liposomal membrane composition has with respect to the chemical nature and the subsequent physical conformation of PS-lipid conjugates. The physical conformation of the PS-lipid conjugate is a direct consequence of its different chemical nature. While both the chemical nature and physical conformation of the PS-lipid conjugate are likely to affect functionality in concert, the physical differences can dictate a lot of their behavior, namely: (1) photochemical efficiency following self-assembly and various degrees of static quenching, (2) accessibility of RMS to the biological milieu, (3) accessibility of probes to RMS that reach beyond the bilayer, (4) intracellular uptake, (5) serum corona size and composition, (6) tumor delivery, and (7) tumor selectivity, amongst others. In this study, we explore the collective functional impact of liposome membrane composition using *in vitro* and *in vivo* orthotopic human head and neck cancer models, in addition to fundamental photophysical and photochemical analyses.

While we have previously reported liposomes containing DSPE-PEG-BPD, the hydrophobicity of BPD prevents it from assuming a truly membrane-protruding conformation. When included at quantities exceeding 0.15 mol%, the liposomes aggregate immediately the following extrusion as a result of hydrophobic interactions between the DSPE-PEG-BPD containing liposomes [21]. Even at 0.15 mol% DSPE-PEG-BPD, the liposomes are 50% larger than BPD-PC liposomes making them unsuitable as counterparts when exploring the role of conformation also. The larger size is likely a result of membrane contortion as the BPD reinserts into the bilayer upon lipid film. The opposite is also true that a strongly hydrophilic PS molecule cannot be stably inserted into the bilayer just by conjugating it to PC. Even with amphiphilic PS molecules such as indocyanine green (ICG), a truly membrane-protruding conformation is not possible. A salient study by Lajunen et al. has demonstrated that ICG can be stably inserted into the bilayer when mixed during lipid film preparation, and can also noncovalently be hidden within the PEG brush if introduced to the liposomes after self-assembly [28]. This important study explored the fundamental functional impact of the membrane localization of ICG i.e. entrapped in lipid bilayer versus being noncovalently inserted in the PEG brush. While the study did demonstrate that the location of ICG resulted in marked differences in photochemistry, phototriggered calcein release, and PS stability, the study did not explore the impact on phototoxicity, tumor kinetics, or pharmacokinetics *in vivo*. Importantly, the study did not use a PS-lipid conjugate, and thus did not probe the functional impact of both the nature and conformation of a PS-lipid conjugate in the membrane.

**Figure 1:**
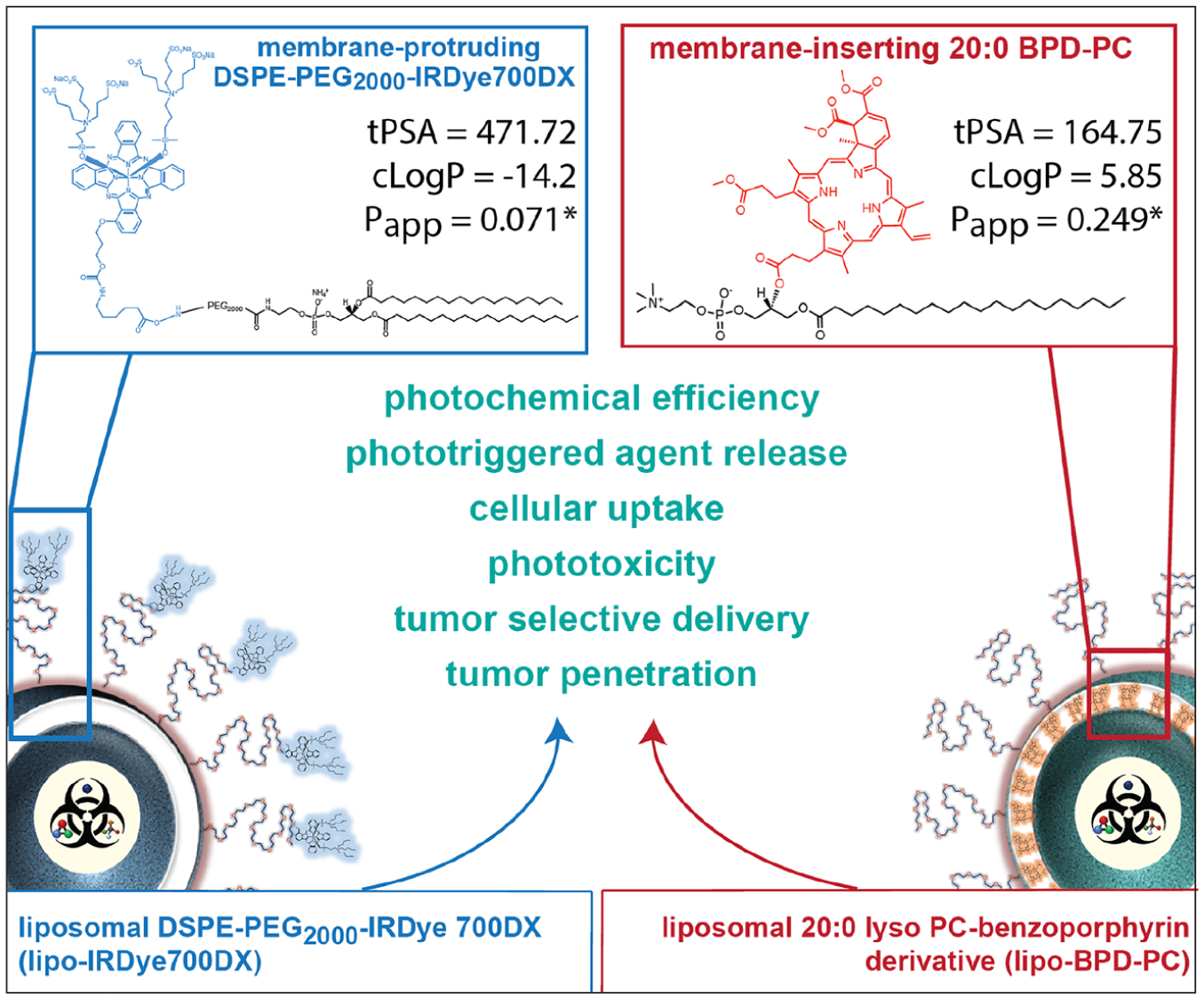
Graphical representation of liposomal photonanomedicines formulated with membrane-protruding DSPE-PEG-IRDye 700DX or membrane-inserting 20:0 lyso-PC-benzoporphyrin derivative (BPD-PC). Topological polar surface area (tPSA) and calculated octanol/water partitions (cLogP) of the PS molecules were determined using ChemDraw 18.0. The apparent biomembrane permeability coefficients (*P*_app_) were simulated using pkCSM computational modeling [29].(* depicts values ×10^−6^ cm/s).

The physical conformation of a PS-lipid conjugate is primarily a consequence of its chemical nature. Considering that a single PS molecule cannot simultaneously be stably inserting into the bilayer and stably protruding away from the bilayer, it is imperative that two separate PS molecules are used to conjugate to lipids. This is to achieve both discrete membrane-inserting and membrane-protruding conformations, given that the differences in the chemical nature between the two are understood and accounted for. As such, here we report the synthesis of a novel membrane-protruding DSPE-mPEG_2000_-NH_2_ conjugate of IRDye700DX, a hydrophilic clinical PS in phase III clinical trials for photoimmunotherapy of head and neck cancer (Clinical-Trials.gov Identifier: NCT03769506; accessed Mar 2021). Liposomes containing the membrane-protruding DSPE-mPEG_2000_-IRDye700DX are contrasted with liposomes containing the membrane-inserting 20:0 lyso PC-BPD conjugate (Figure 1). The membrane-protruding and membrane-inserting conformations are validated by computational predictions of their topological polar surface area (tPSA; ChemDraw 18.0), calculated octanol/water partitions (cLogP; ChemDraw 18.0), and apparent biomembrane permeability coefficients (*P*_app_), as well as photophysical analysis. The two membrane compositions are evaluated in terms of their impact on photochemical efficiency, cellular uptake, subcellular localization, PDT efficacy, phototriggered and passive agent release kinetics, *in vivo* pharmacokinetics, and tumor-selective delivery in orthotopic FaDu head and neck cancer murine xenografts. To the best of our knowledge, this is the first demonstration of how the membrane composition of NIR-activable liposomes containing PS-lipid conjugates is a key determinant of their functionality as photodynamic and phototriggerable platforms for tumor-selective multi-agent therapy. As such, our results enable the rational design of NIR-activable liposomes by introducing membrane composition (both chemical nature and subsequent physical conformation of PS-lipid conjugates) as a critical component that can be selected in a purpose-specific manner for optimal therapeutic and theranostic functionality. Furthermore, combinations of both membrane compositions we demonstrate here could be rationally integrated into a single construct to capitalize on the powerful properties of both.

## 2 Results

### 2.1 Fabrication and characterization of NIR-activable liposomes

In this study, liposomes were fabricated as a prototypical lipid-based PNM platform, which comprised 0.5 mol% of either a novel membrane-protruding DSPE-PEG_2000_-NH_2_ lipid conjugate of IRDye700DX or a membrane-inserting 20:0 lyso-PC lipid conjugate of BPD which we have previously reported (Figure 1) [18, 21]. The membrane-protruding and membrane-inserting conformations of IRDye 700DX and BPD, respectively, are primarily dictated by the inherent chemical differences between the two molecules. The membrane-protruding and membrane-inserting conformations are then conferred by conjugating IRDye 700DX to the hydrophilic membrane-protruding terminal amine of DSPE-PEG_2000_-NH_2_ and by conjugating BPD to the membrane-inserting sn-2 hydroxyl group of 20:0 lyso PC, respectively. Computational predictions of the membrane conformation of the two PS molecules were simulated and validated by their topological polar surface area (tPSA; ChemDraw 18.0), calculated octanol/water partitions (cLogP; ChemDraw 18.0), and apparent biomembrane permeability coefficients (*P*_app_). *P*_app_ was simulated using the pkCSM model that predicts small-molecule pharmacokinetic properties using graph-based signatures [29]. Simplified molecular-input line-entry system sequences of IRDye 700DX and BPD were obtained from PubChem (https://pubchem.ncbi.nlm.nih.gov) and were used for the pkCSM model. Computational simulations predicted that DSPE-PEG-IRDye700DX was in fact membrane protruding, with a high topological polar surface area (tPSA) of 471.72, a low cLogP of −14.2 and a low biomembrane permeability coefficient of *P*_app_ = 0.071 × 10^−6^ cm/s. Computational simulations also predicted that the 20:0 Lyso PC-BPD (abbreviated to BPD-PC) was in fact membrane-inserting, with a 2.9-fold lower tPSA of 164.75, a higher cLogP of 5.85 and a 3.5-fold higher biomembrane permeability coefficient of *P*_app_ = 0.249 × 10^−6^ cm/s (Figure 1).

**Figure 2:**
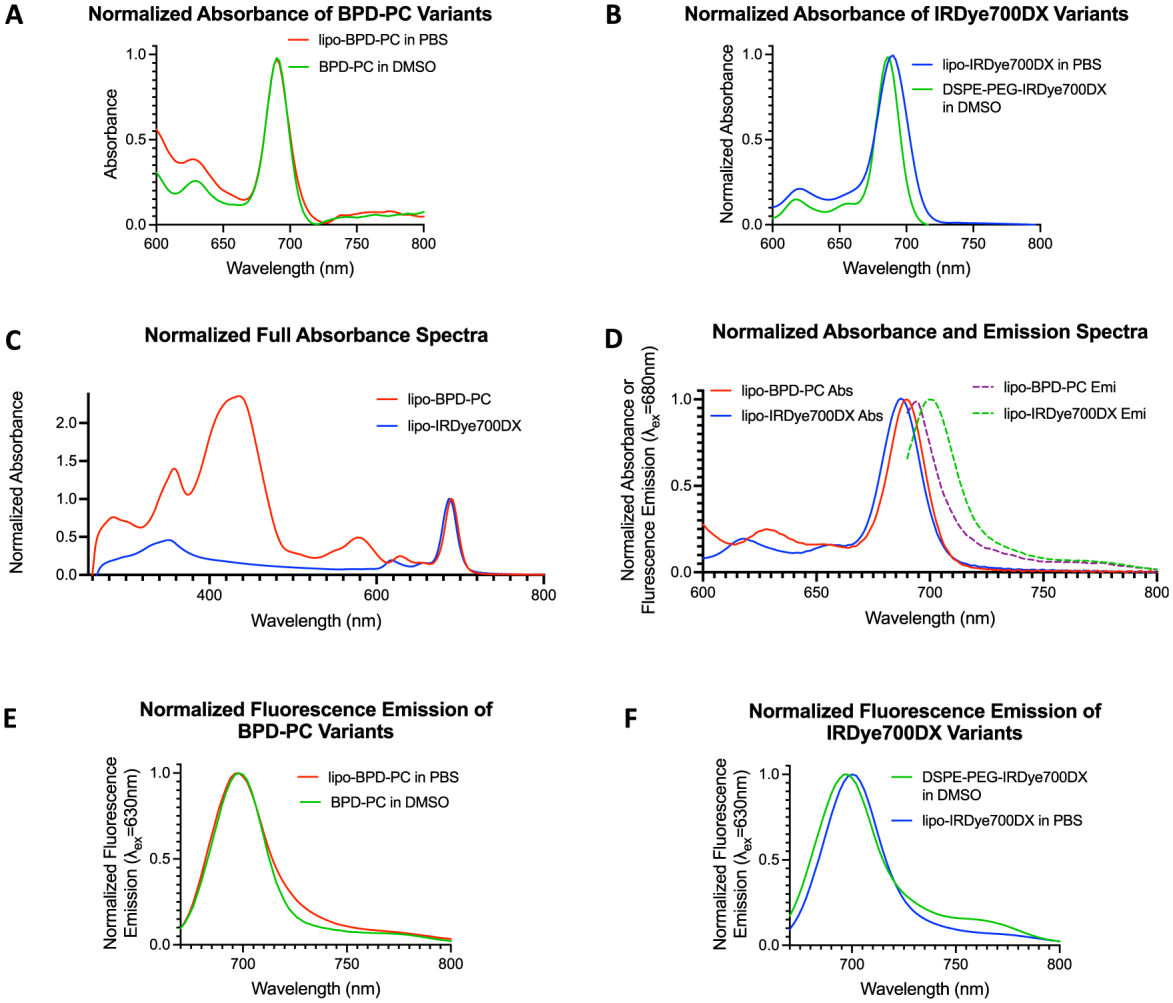
Normalized absorbance spectra of BPD-PC in free form and in liposomal form (A) and of DSPE-PEG-IRDye700DX in free form and in liposomal form (B). (C) Normalized full absorbance spectra of both liposomes reveal the Soret and Q-bands of both PSs. (D) Normalized absorbance and emission spectra (Exc_680 nm_) of both liposomes at the red-NIR region. Normalized fluorescence emission spectra of BPD-PC in free form and in liposomal form (E) and of DSPE-PEG-IRDye700DX in free form and in liposomal form (F) using Exc_630 nm_ to reveal the full emission band profiles and to simulate the excitation of the *in vivo* fluorescence imaging system.

**Figure 3:**
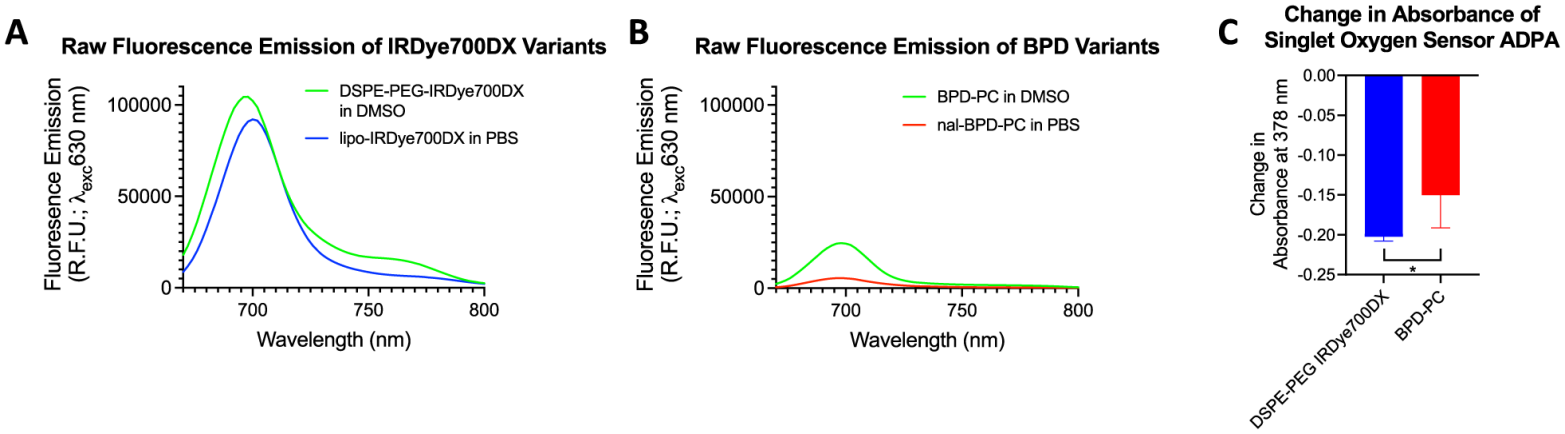
Raw fluorescence emission spectra of DSPE-PEG IRDye700DX and lipo-IRDye700DX (A) and of BPD-PC and lipo-BPD-PC (B) using Exc_630 nm_ to reveal the full emission band profiles and to simulate the excitation of the *in vivo* fluorescence imaging system. (C) Singlet oxygen production by the unformulated lipid conjugates of IRDye700DX and BPD in DMSO detected by a decrease in absorbance of the colorimetric singlet oxygen probe, anthracene dipropionic acid (ADPA; maximum peak centered at 378 nm). BPD-PC produces 25% less singlet oxygen than DSPE-PEG IRDye700DX.

The absorption spectra of each PS-lipid conjugate and respective liposomal formulations are shown in Figure 2A and B. Lipidated BPD-PC exhibited an identical absorbance when free in dimethyl sulfoxide (DMSO) and when formulated into liposomes in phosphate buffered saline (PBS) (Figure 2A). However, DSPE-PEG-IRDye700DX exhibited a 4 nm red shift in the Q-band absorbance maximum when formulated into liposomes in PBS (Figure 2B). The full absorbance spectra showing the Soret and Q-bands of lipo-IRDye700DX and lipo-BPD-PC are shown in Figure 2C. The Q-band absorbance and emission (Exc_680 nm_) spectra of lipo-IRDye700DX and lipo-BPD-PC are shown in Figure 2D. As expected, the Q-band emission maximum of BPD-PC was unchanged following lipid conjugation (Figure 2E). However, the Q-band emission maximum of DSPE-PEG-IRDye700DX was red-shifted by 2 nm upon liposomal formulation (Figure 2F), which corresponded to the slight red-shifting observed in the Q-band absorbance maximum. While the conjugation of 20:0 lyso PC conjugation to BPD had no impact on its fluorescence intensity in DMSO, conjugation of IRDye700DX to DSPE-PEG_2000_-NH_2_ enhanced its fluorescence emission by 15.1% in DMSO (Figure S1A and B and Table 1). However, upon liposomal formulation, the intensities of the fluorescence emission of the PS-lipid conjugates changed significantly as a result of varying degrees of static quenching, a phenomenon that is influenced by their membrane-protruding and membrane-inserting conformations (Figure 3A and B). The membrane-protruding lipo-IRDye700DX exhibits a moderate 25% fluorescence quenching upon formulation, whereas membrane-inserting lipo-BPD-PC exhibits a 3-fold greater quenching at 74.2% upon formulation (Figure 3A and Table 1). This marked difference is likely due to the tight packing of membrane-inserting lipo-BPD-PC, which is unlike the less-dense PS packing in the membrane-protruding lipo-IRDye700DX. The Q-band emission spectra of lipo-IRDye700DX and lipo-BPD-PC following excitation of the Soret bands (Exc_354 nm_) are shown in Figure S1C.

The only clinically used form of IRDye700DX is the clinical photoimmunoconjugate Cetuximab (Cet)-IRDye700DX. As such, Cet-IRDye700DX, as well as free PS molecules have been used as controls in this study. Lipo-IRDye700DX and Cet-IRDye700DX have identical absorbance and emission profiles; however, Cet-IRDye700DX exhibits 3-fold greater quenching of fluorescence emission likely due to increased static quenching when conjugated to the antibody (Figure S1D–F) [30].

The hydrodynamic diameters of the lipo-IRDye700DX and lipo-BPD-PC were found to be 144.3 and 138.1 nm, respectively (Table 2). Matching of hydrodynamic diameters is critical for eliminating size-based differences in cellular uptake, cellular phototoxicity, tumor delivery, tumor tissue selectivity, and tumor penetration. The *ζ*-potential of the lipo-IRDye700DX was 5.85 mV less than that of the lipo-BPD-PC, which could be attributed to the 6 sulfonates per IRDye700DX molecule at the surface of the lipo-IRDye700DX (Table 2). Although this difference is almost negligible, it is taken into consideration throughout the study. While the nature of the two PSs is the most pronounced *chemical* difference between the membrane-protruding lipo-IRDye700DX and membrane-inserting lipo-BPD-PC, the conformation of the PS-lipid conjugates remains to be the most pronounced *physical* difference between the two. It is also important to note that lipo-IRDye700DX exhibits a 15.3-fold greater fluorescence intensity than lipo-BPD-PC in PBS (Figure 3A and B), which has implications in theranostic tumor imaging. This is also consistent in serum (Figure S2). While naturally, the two PSs are inherently different in their photochemical efficiency and singlet oxygen quantum yields, upon 690 nm light-emitting diode (LED) illumination in DMSO, unformulated BPD-PC appears to be only 25% less efficient than unformulated DSPE-PEG-IRDye 700DX, as demonstrated by decay in absorbance of the singlet oxygen probe anthracene dipropionic acid (ADPA; Figure 3C). In addition to the fact that both BPD and IRDye 700DX are activatable by the same wavelength of NIR light, this small difference in their photochemical efficiency makes the two sensitizers appropriate for the comparison of the two conformations given that differences in their chemical nature are taken into consideration. Their chemical natures must be different in order to allow the two liposomes to exhibit either a truly membrane-protruding conformation or membrane-inserting conformation. These chemical differences are taken into consideration throughout the study and are further discussed in the respective results sections.

### 2.2 Reactive molecular species generation

**Figure 4:**
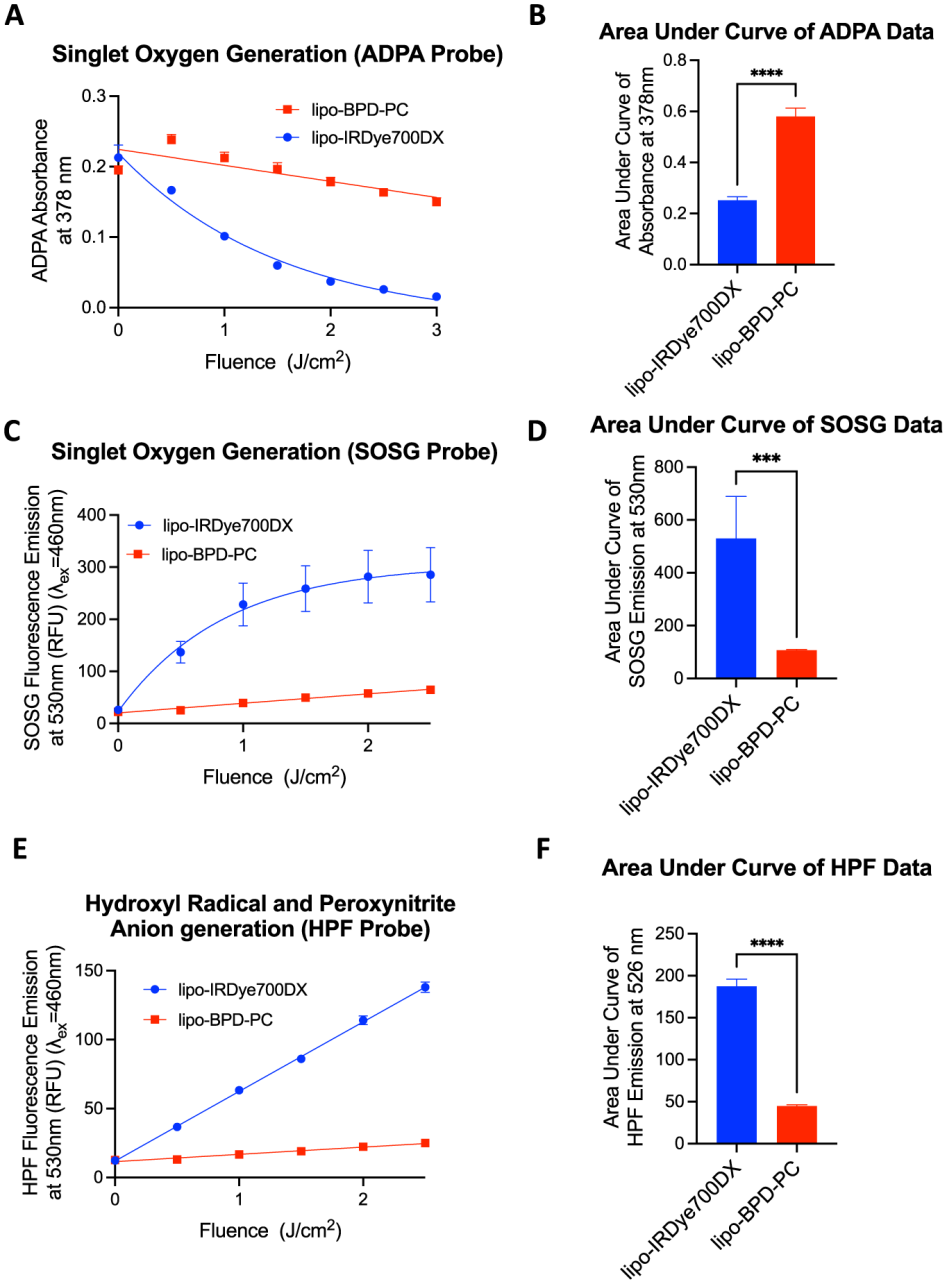
(A and B) singlet oxygen generation by the membrane-protruding lipo-IRDye700DX and membrane-inserting lipo-BPD-PC in PBS measured by the decreasing absorbance of the colorimetric singlet oxygen probe anthracene dipropionic acid (ADPA). (C and D) singlet oxygen generation by the two formulations measured by the increasing fluorescence emission of singlet oxygen sensor green (SOSG). (E and F) hydroxyl radical and peroxynitrite anion generation by the two formulations measured by the increasing fluorescence emission of hydroxyphenyl fluorescein (HPF). (Data are mean ± standard error; statistical significance was calculated using a two-tailed *t*-test, ***: *P* ≤ 0.0002, ****: *P* ≤ 0.0001).

We have previously shown that fluorescence quenching of lipidated PS molecules upon formulation into liposomes and micelles directly correlated with a reduction in singlet oxygen generation [21]. As such, we evaluated the additional impact that conformation has on the photochemical efficiencies of the membrane-protruding lipo-IRDye700DX and membrane-inserting lipo-BPD-PC given the aforementioned 25% difference between the two. Photochemical efficiency was assessed using three optochemical techniques. Singlet oxygen production following 690 nm irradiation was measured using the colorimetric probe anthracene dipropionic acid (ADPA) (photobleaches upon oxidation) as described earlier and using the fluorometric probe Singlet Oxygen Sensor Green (SOSG). Furthermore, photochemical production of hydroxyl radicals and peroxynitrite radicals was measured using the fluorometric probe hydroxyphenyl fluorescein (HPF). These measurements were all performed while taking into consideration that the efficiency of singlet oxygen production by DSPE-PEG-IRDye700DX is inherently *ca*. 25% greater than that of BPD-PC. Results reveal that the membrane-protruding lipo-IRDye700DX is consistently more efficient than membrane-inserting lipo-BPD-PC at the photogeneration of singlet oxygen, hydroxyl radicals, and peroxynitrite radicals upon 690 nm LED photoexcitation (Figure 4). With regards to the photogeneration of singlet oxygen as measured by ADPA, lipo-IRDye700DX is 2.3-fold more efficient than lipo-BPD-PC (Figure 4A and B). This difference exceeds the inherent *ca*. 25% (1.25-fold) greater efficiency in singlet oxygen production of DSPE-PEG-IRDye700DX. This suggests that the lower fluorescence quenching seen in the membrane-protruding lipo-IRDye700DX is largely responsible for its superior efficiency in singlet oxygen production. When using SOSG, the singlet oxygen production of lipo-IRDye700DX is found to be 4.9-fold more efficient than for lipo-BPD-PC (Figure 4C and D). Similarly, the hydroxyl radical and peroxynitrite radical photogeneration was found to be 4.2-fold more efficient for the membrane-protruding lipo-IRDye700DX, as compared to the membrane-inserting lipo-BPD-PC (Figure 4E and F). It is likely that the lower photochemical efficiencies observed in the membrane-inserting lipo-BPD-PC predominantly also a result of conformational differences between the two liposomes. It is conceivable that the probes ADPA, SOSG, and HPF are also less accessible to the membrane-inserted BPD-PC than to the membrane protruding DSPE-PEG-IRDye700DX, hence detecting less RMS. The opposite may also be true that the RMS are not capable of traveling far enough away from the membrane to be detectable by the probes ADPA, SOSG, and HPF. This would also be true for the accessibility of RMS to the immediate biological environment. In either case, the PS-lipid conjugate conformation here appears to be the predominant factor influencing their photochemical efficiencies.

As controls, the photochemical efficiency of free IRDye700DX and the clinical photoimmunoconjugate Cet-IRDye700DX were also measured (Figure S3). While the photochemical efficiency of free IRDye700DX was marginally greater than that of lipo-IRDye700DX, lipo-IRDye700DX was significantly more efficient than the clinical photoimmunoconjugate Cet-IRDye700DX. As compared to Cet-IRDye700DX, this superior photochemical efficiency seen in the lipo-IRDye700DX can also be attributed to the significant 76.43% fluorescence quenching observed in Cet-IRDye700DX (Figure S1).

### 2.3 Passive and phototriggered calcein release

**Figure 5:**
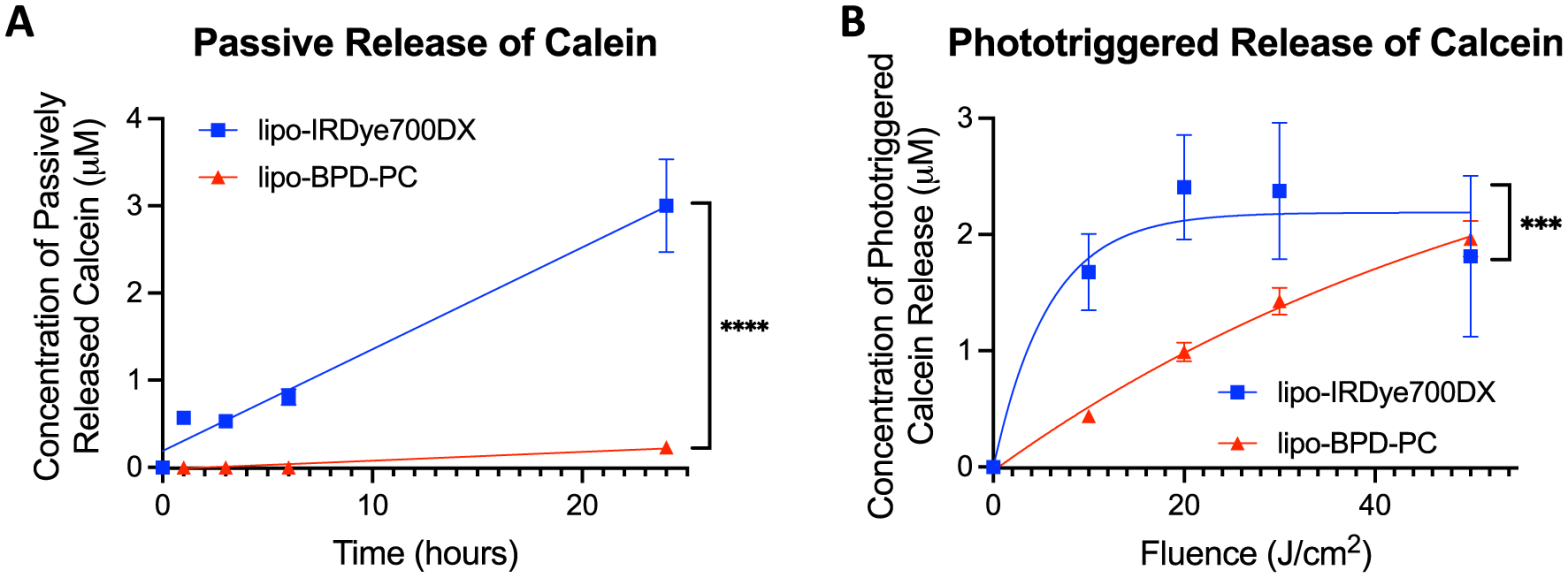
Phototriggered (A) and passive (B) calcein release from lipo-IRDye700DX and lipo-BPD-PC. Phototriggered calcein release was performed using 690 nm light at an irradiance of 17.86 mW cm^−2^ (Data are mean ± standard error; statistical significance was calculated using one-way ANOVA with a Tukey post-test, ***: *P* < 0.001; ****: *P* < 0.0001).

The purpose of photochemistry for NIR-activable liposomes and PNM platforms, in general, is two-fold: (1) to mediate photodynamic cancer cell killing, and (2) for phototriggered drug release to spatiotemporally control induction of combination regimens. As such, we evaluated the efficiency of phototriggered release of a hydrophilic drug surrogate, namely, calcein disodium salt, encapsulated in the core of lipo-IRDye700DX and lipo-BPD-PC at a self-quenching concentration of 100 mM. Dequenching of calcein fluorescence was used as a prototypical indicator of agent released from the liposomes. While both liposomes ultimately released comparable levels of calcein upon irradiation with a total of 50 J cm^−2^ (2 μM; 40% of entrapped calcein), the rate of phototriggered release of calcein from lipo-IRDye700DX (*k* = 0.201/J cm^−2^) was 11.92-fold greater than that of the lipo-BPD-PC (*k* = 0.0169/J cm^−2^) (Figure 5A). The membrane-protruding lipo-IRDye700DX elicited a burst-release of calcein, while the membrane-inserting lipo-BPD-PC mediated a steadier, sustained phototriggered release of calcein. In addition, membrane composition also influenced the passive release of calcein at 37 °C over the span of 24 h. Results show that lipo-BPD-PC passively releases calcein at a rate that is 13.2-fold slower than lipo-IRDye700DX in the dark (Figure 5B). By 24 h, 3 μM calcein (60%) was passively released from the membrane-protruding lipo-IRDye700DX, whereas only 0.23 μM calcein (4.5%) was passively released from the membrane-inserting lipo-BPD-PC. As such, it can be concluded that the presence of membrane-inserting BPD-PC significantly reduces the liposomal membrane permeability of entrapped molecules. A similar trend has been previously observed, whereby liposome membrane permeability was reduced by more than 10-fold when a membrane-inserting porphyrin-lipid conjugate was included in the bilayer [31]. A reduction in membrane permeability by the membrane-inserting BPD-PC is also likely to decrease the susceptibility of the membrane to phototriggered release [31]. These findings suggest that the membrane-protruding lipo-IRD ye700DX, is better suited as a rapid burst release platform for entrapped agents, whereas the membrane-inserting lipo-BPD-PC, is better suited for applications whereby controlled and sustained agent release is required. Interestingly, the results also suggest that membrane insertion of PSs is not necessary for phototriggered agent release from liposomes, as the nearby localization of the membrane-protruding IRDye700DX is sufficient and in fact superior for phototriggered release. This is consistent with the key study by Lajunen et al. which showed that ICG noncovalently associated with the PEG coating of a liposome was still capable of inducing phototriggered calcein release [28]. Furthermore, it appears that release kinetics are likely to be dictated by a combination of the photochemical efficiency of the system and by passive membrane permeability, which appears to be significantly reduced by the presence of the membrane-inserting BPD-PC lipid conjugate (Figure 5B).

### 2.4 Cellular uptake of liposomes and PS variants

**Figure 6:**
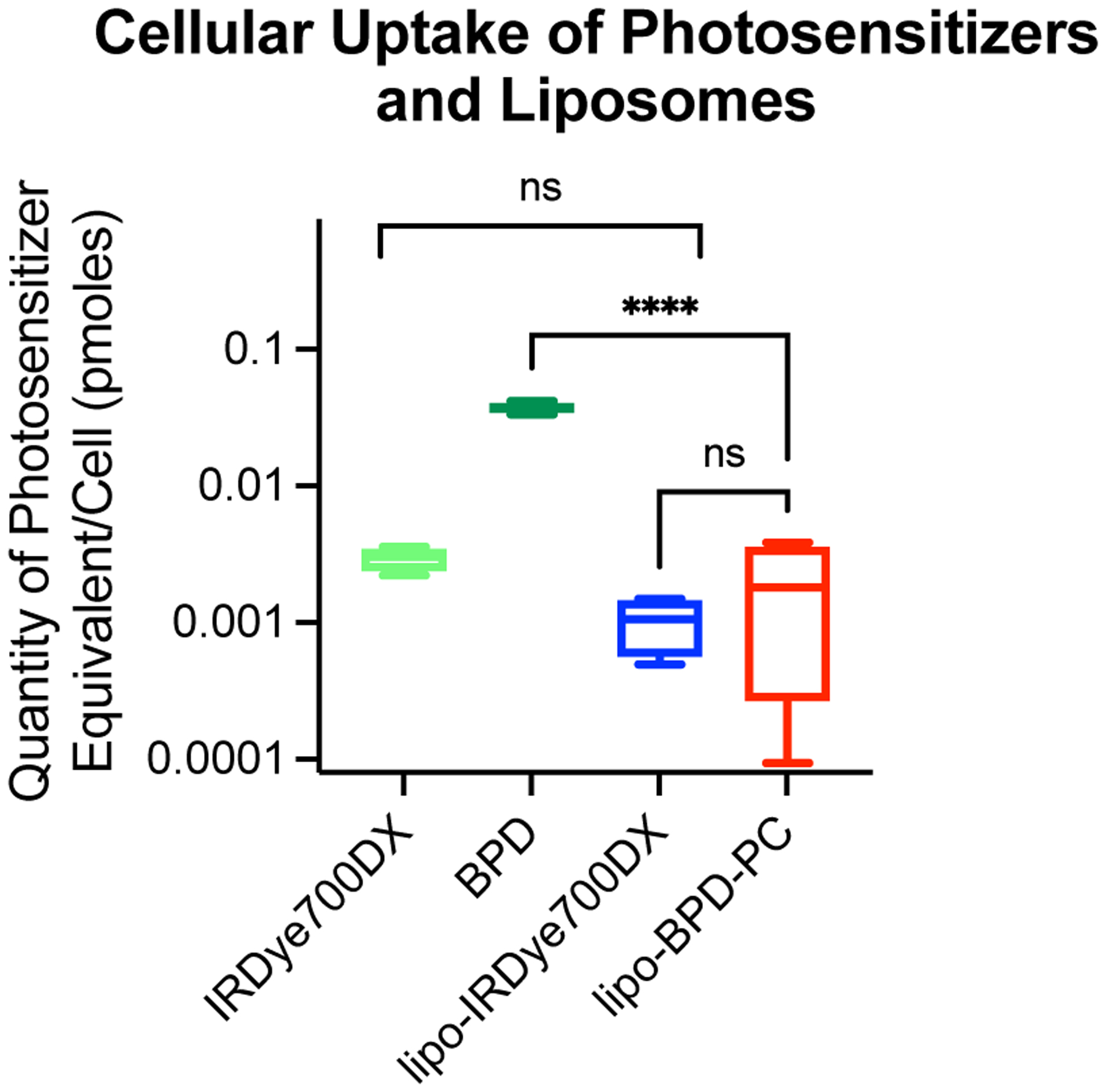
Comparison of FaDu cell uptake of lipo-IRDye700DX and lipo-BPD-PC, as well as free IRDye700DX and free BPD after 24 h incubation. (Data are mean ± standard error; statistical significance was calculated using one-way ANOVA with a Tukey post-test, ****: *P* ≤ 0.0001).

Cellular interaction and uptake are oftentimes key determinants for the efficacy of liposomes as PDT agents and as nanoplatforms for combination therapy. As such, we explored the impact of membrane composition of the membrane-protruding lipo-IRDye700DX and membrane-inserting lipo-BPD-PC in FaDu human head and neck cancer cells. It was found that no significant difference existed between the cellular uptake of lipo-IRDye700DX and lipo-BPD-PC, suggesting that the small difference in *ζ*-potential also had no impact on internalization (Figure 6). Interestingly, while there was no significant difference in uptake between the free IRDye700DX and the lipo-IRdye700DX, the Cet-IRDye700DX clinical photoimmunoconjugate control was internalized more efficiently (Figure S4). This can be explained by the receptor-mediated endocytosis through targeting of epidermal growth factor receptor (EGFR) by Cetuximab. Owing to its small molecular weight and amphiphilic nature, free BPD was the most efficiently internalized agent tested. These results thus suggest that both the membrane-protruding lipo-IRDye700DX and membrane-inserting lipo-BPD-PC present themselves as equivalent intracellular drug delivery systems.

### 2.5 Intracellular localization

**Figure 7:**
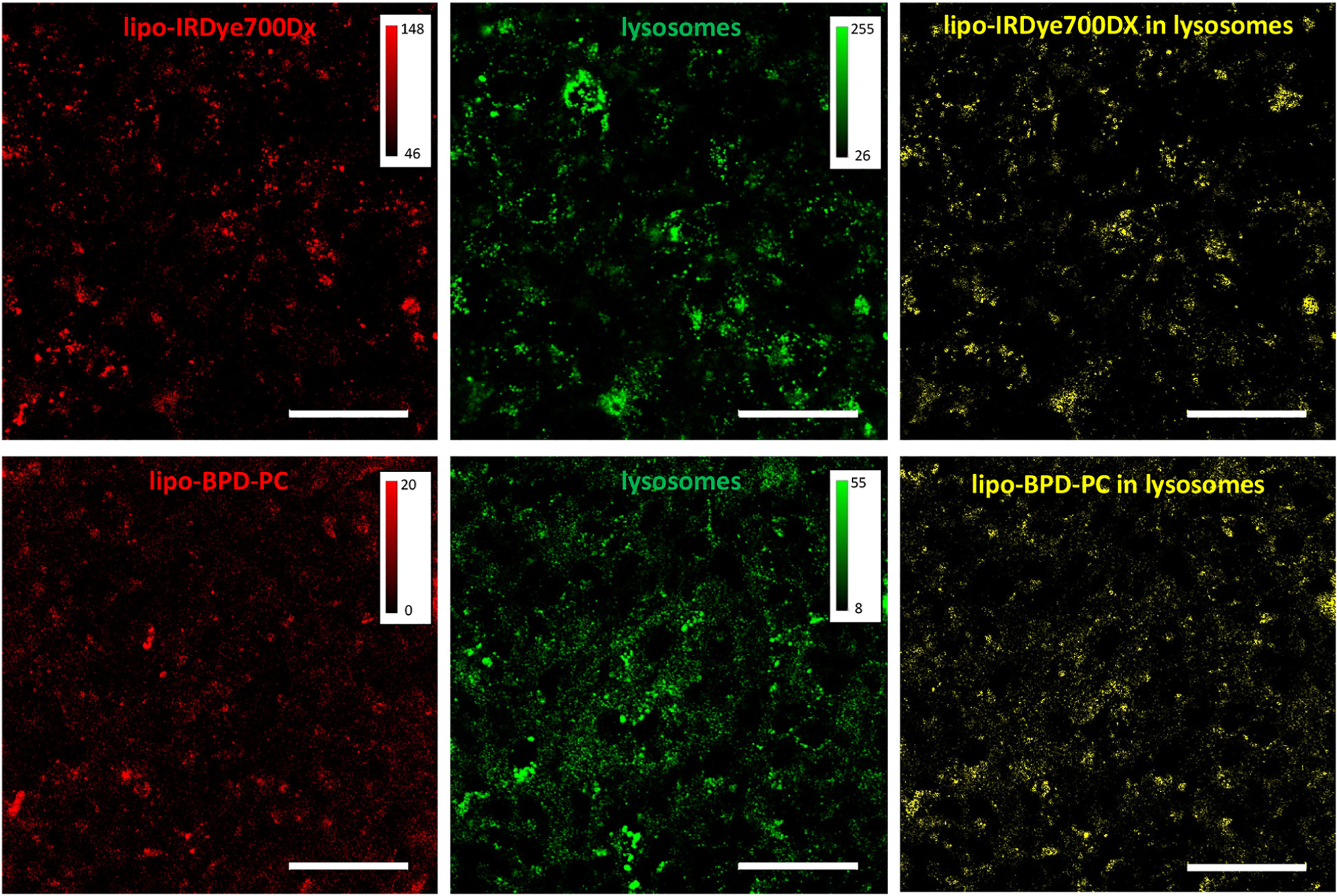
Confocal microscopy images of FaDu cells at 60× magnification after a 24 h incubation with lipo-IRDye700DX (top, red), lipo-BPD-PC (bottom, red), and LysoTracker^™^ Green DND-26 (middle, green). The liposomes co-localized with the lysosomes are presented in the processed logical AND operator images (right, yellow, processed on ImageJ). (Scale bars are 50 μm).

Although the degree of FaDu cell uptake was equivalent for both liposomes, we further explored whether the constructs were localized in different organelles. Our previous findings have shown that various iterations of lipo-BPD-PC strictly localize within endolysosomes [18–20]. This is not atypical, as endocytosis of liposomes leading to their presence in endolysosomal compartments has been widely reported for various types of liposomes [32–34]. In this study, we incubated FaDu cells with both liposomes for 24 h, and the cells were counter-stained with LysoTracker^™^ Green DND-26 prior to imaging with confocal microscopy. Microscopy images (Figure 7) revealed that both lipo-BPD-PC and lipo-IRDye700DX appear as puncta within cells, which is typical of sequestration in endolysosomal compartments. This was further confirmed by the partial colocalization of both lipo-BPD-PC and lipo-IRDye700DX with LysoTracker^™^ Green DND-26. Images of lipo-BPD-PC and lipo-IRDye700DX were processes with the corresponding the LysoTracker^™^ Green DND-26 images to isolate pixels that were co-localized, thereby demonstrating the liposomes that were present within the endolysosomal compartments (yellow, Figure 7). These results confirm that both liposome compositions result in the same localization within endolysosomal compartments in FaDu cells, and are internalized with the same efficiency as demonstrated earlier. This is not unexpected considering that the sizes of both liposomes are matched, and also suggests that the membrane composition along with the small difference in *ζ*-potential have no impact on their intracellular trafficking.

### 2.6 Photodynamic therapy

**Figure 8:**
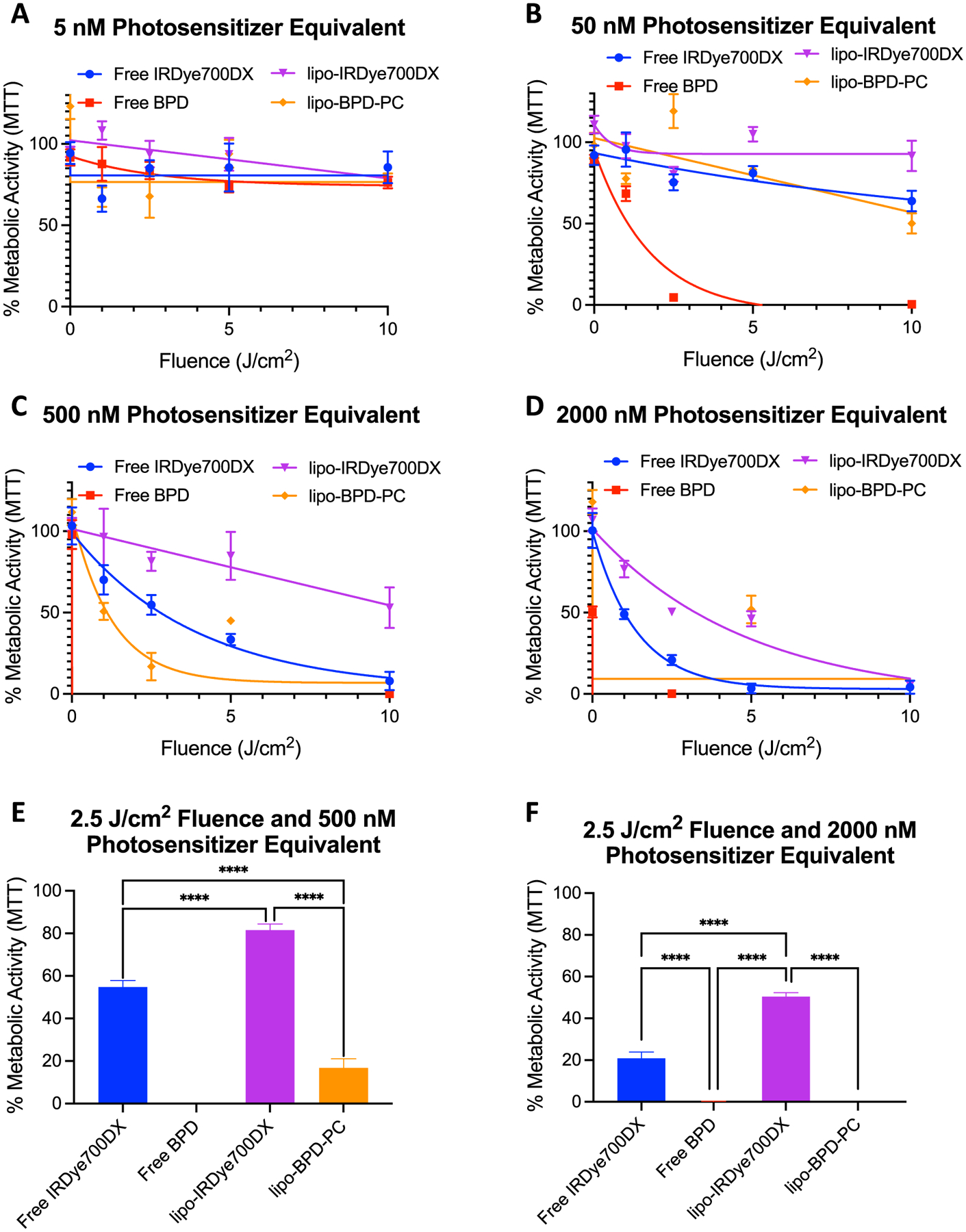
Percent metabolic activity of FaDu cells as determined by the MTT assay performed 48 h following PDT with lipo-IRDye700DX, lipo-BPD-PC, and free PS controls. Concentrations were at 5 nM (A), 50 nM (B), 500 nM (C), and 2000 nM (D) PS equivalent and irradiation at 690 nm light was performed at an irradiance of 27.7 mW cm^−2^ up to a fluence of 10 J cm^−2^. The percent metabolic activity at a fluence of 2.5 J cm^−2^ with a PS equivalent of 500 nM (E) and 2000 nM (F) is also presented. (Data are mean ± standard error; statistical significance was calculated using one-way ANOVA, ****: *P* ≤ 0.0001).

As we have previously shown that photochemical efficiency alone is not prognostic of *in vitro* photodynamic efficacy of lipid-based PNMs (liposomes as well as micelles) [21], we further investigated the role of lipo-IRDye700DX and lipo-BPD-PC membrane composition on FaDu cell phototoxicity. Despite being 2–5-fold less effective at generating RMS, lipo-BPD-PC was significantly more effective than lipo-IRDye700DX at photodestruction of FaDu cells (Figure 8). At 500 nM PS equivalent, lipo-BPD-PC (LD_50_ = 1.1 J cm^−2^) was 9.9-fold more phototoxic than lipo-IRDye700DX (LD_50_ = 10.9 J cm^−2^). At 2000 nM PS equivalent, lipo-BPD-PC was also significantly more effective than lipo-IRDye700DX at cell killing, as the LD_50_ of lipo-IRDye700DX was 3.25 J cm^−2^, while the LD_50_ of lipo-BPD-PC was too low to be accurately determined (Figure 8E and F). Interestingly, the superior phototoxicity of the lipo-BPD-PC cannot be attributed to a difference in the efficiency of cellular internalization, subcellular localization, or to its photochemical efficiency, which remains 2–5-fold less efficient than lipo-IRDye700DX. It is unclear at this stage what the cause for this enhanced phototoxicity is, yet it is most plausible that the radical-based photodegradation products of lipo-BPD-PC have a greater impact on cell viability than those generated by the photo-irradiation of lipo-IRDye700DX. This hypothesis will be the focus of key future mechanistic studies. Of all agents tested, the Cet-IRDye700DX control was the most cytotoxic (Figure S5). Its cytotoxicity, however, was greatly influenced by its dark toxicity, which can be attributed to EGFR downregulation and subsequent induction of apoptosis [35].

### 2.7 In vivo tumor kinetics and pharmacokinetics

**Figure 9:**
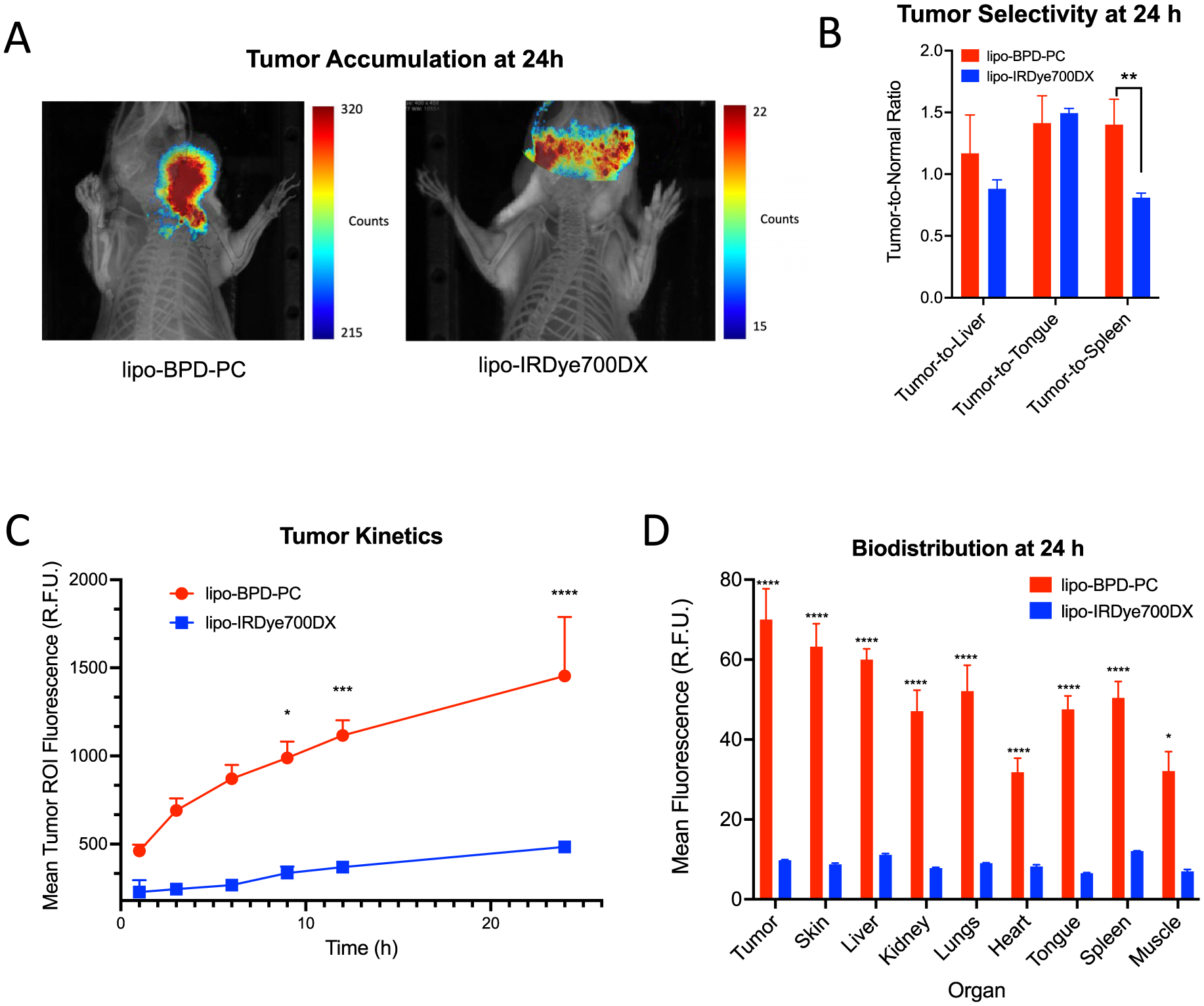
(A) Representative FLI/μCT images of orthotopic FaDu tumors 24 h following intravenous administration of lipo-BPD-PC and lipo-IRDye700DX. (B) Tumor selectivity of lipo-BPD-PC and lipo-IRDye700DX 24 h following intravenous administration. (C) Longitudinal fluorescence imaging of lipo-IRDye700DX and lipo-BPD-PC in orthotopic FaDu tumors to assess tumor kinetics. (D) Semi-quantitative fluorescence-based analysis of liposome biodistribution 24 h following intravenous administration. (Data are mean ± standard error; *n* ≥ 4 mice per arm; statistical significance was calculated using one-way ANOVA with a Tukey post-test, *: *P* ≤ 0.0.5, **: *P* ≤ 0.005, ***: *P* ≤ 0.0005, ****: *P* ≤ 0.0001).

Finally, the impact of membrane composition on *in vivo* pharmacokinetics and tumor penetrating behavior was studied in an orthotopic murine model of FaDu human head and neck cancer. Swiss nu/nu mice bearing FaDu tumors that were implanted by a transcervical injection through the floor of the mouth were injected intravenously with 6.95 nmol PS equivalent of the membrane-protruding lipo-IRDye700DX or membrane-inserting lipo-BPD-PC. Interestingly, tumor accumulation was visibly more efficient at 24 h for the membrane-inserting lipo-BPD-PC than for the membrane-protruding lipo-IRDye700DX (Figure 9A). At 24 h, the tumor selectivity of the lipo-BPD-PC was also significantly higher than that of the lipo-IRDye700DX (Figure 9B). Using semi-quantitative fluorescence-based analysis of liposome tumor kinetics, tumor accumulation of the lipo-BPD-PC was found to be higher than that of the lipo-IRDye700DX at all time points within 24 h following administration (Figure 9C). Furthermore, biodistribution analysis of tumor uptake revealed that tumor uptake of lipo-BPD-PC was 7.16-fold greater than the lipo-IRDye700DX at 24 h following administration (Figure 9D). It was also found that tissue uptake of lipo-BPD-PC was higher than that of lipo-IRDye700DX at 24 h following administration in all other organs assessed.

**Figure 10:**
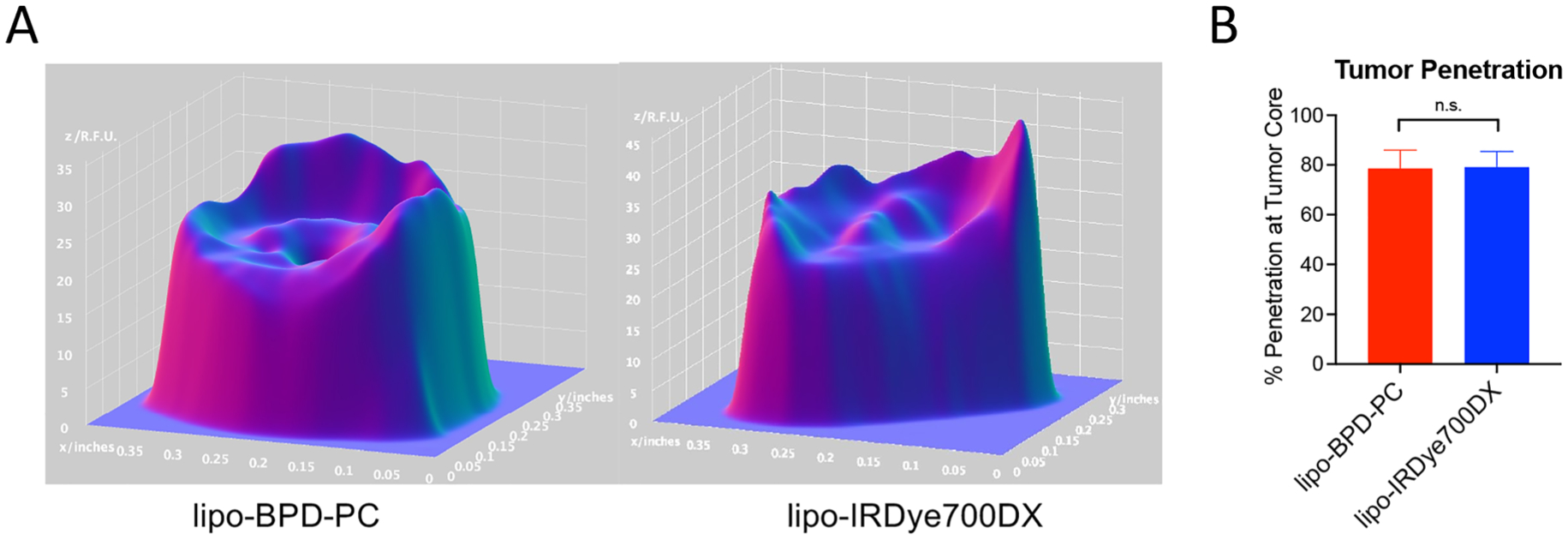
(A) Three-Dimensional projections of tumor penetration of lipo-IRDye700DX and lipo-BPD-PC in orthoptic FaDu tumors 24 h after administration. The *x*- and *y*-axis correspond to spatial dimensions (inches) and the *z*-axis corresponds to the fluorescence intensities of the lipo-IRDye700DX and lipo-BPD-PC (R.F.U). (B) Quantitation of tumor penetration at the tumor core reveals no difference between lipo-IRDye700DX and lipo-BPD-PC. (Data are mean ± standard error; *n* ≥ 4 tumors per arm, statistical significance was calculated using one-way ANOVA with a Tukey post-test).

Although tumor kinetics and *in vivo* pharmacokinetics were markedly altered by the membrane composition, it was interesting to observe that it had no impact whatsoever on tumor penetration. At the core of the tumors, both the lipo-IRDye700DX and the lipo-BPD-PC accumulated at *ca*. 80% with respect to the regions of highest accumulation at the tumor periphery (Figure 10). This is likely due to the fact that the size of the two constructs is matched, which remains to be a major determining factor in the degree of tumor tissue penetration. It is also worth noting that the small difference in *ζ*-potential between the two liposomes did not influence the degree of tumor penetration.

The pronounced differences in orthotopic FaDu tumor kinetics and biodistribution observed between the membrane-protruding lipo-IRDye700DX and membrane-inserting lipo-BPD-PC reveal an unprecedented impact that membrane composition has on liposomes as NIR-activable theranostic drug delivery platforms. How such a marked difference in liposome *in vivo* behavior is influenced purely by membrane composition remains to be the focal point of future studies. Undoubtedly, a deeper understanding is warranted of the molecular constituents and physical attributes of the protein coronas that form on the membrane-protruding and membrane-inserting liposomes upon contact with serum. These future studies into the protein corona, in addition to the careful and detailed function-specific selection of membrane composition, will inevitably prove to be important for the further advancement of multifunctional liposomes.

## 3 Conclusions

NIR-activable liposomes are emerging as tunable platforms for PDT-based anticancer combination regimens. Lipid conjugates of PS molecules serve as the photodynamic agents for phototoxicity as well as phototriggered release of secondary and tertiary therapeutics. As a result of differences in their chemical nature and lipid conjugation sites, these PS-lipid conjugates take on various conformations at the liposomal membrane, with the PSs being either membrane-protruding or membrane-inserting. While both membrane-protruding and membrane-inserting conformations have been reported, the collective functional role of liposome membrane composition on their *in vitro* and *in vivo* behavior is yet to be fully explored. To the best of our knowledge, this study is the first to report that PS-lipid conjugates are not simply passive spectators that facilitate PDT and phototriggered agent release, but their chemical nature and subsequent physical conformation are in fact a key functional determinant of their *in vitro* and *in vivo* behavior. Results in this study show that membrane composition plays central role in photochemistry, photo-dynamic efficacy, phototriggered agent release kinetics, and orthotopic tumor delivery and selectivity. As such, our findings here propose that liposomes containing a membrane-protruding DSPE-PEG-IRDye700DX PS-lipid conjugate are a superior platform for rapid phototriggered agent release. Conversely, liposomes containing a membrane-inserting BPD-PC PS-lipid conjugate are a better-suited platform for sustained phototriggered agent release and efficient photodynamic tumor cell destruction. Furthermore, membrane-inserting lipo-BPD-PC provides significantly greater tumor delivery and therefore presents itself as a superior platform for tumor delivery of combination agents including the PS-lipid conjugate itself. However, owing to its 12-fold enhanced phototriggered release kinetics and 15-fold brighter fluorescence emission, the membrane-protruding lipo-IRDye700DX is a superior platform for phototriggered drug release in the vascular lumen whereby delivery into the tumor interstitium may not be necessary, and as theranostic platforms for optical tumor imaging and image-guided phototherapy.

The mechanisms underlying the unprecedented functional impact of liposome membrane composition are undeniably multifactorial and revolve around differences in the chemical nature of the PS molecules, in addition to their consequent differences in conformation. As we discussed, tumor delivery and tumor tissue selectivity of liposomes are impacted by the protein corona that spontaneously forms on liposomes, as is also the case for nanomedicines in general. Future studies, likely revolving around proteomics, will help identify the differences between the composition of the protein coronas that form on the membrane-inserting lipo-BPD-PC and on the membrane-protruding lipo-IRD ye700DX. These findings may also aid in further advancing nanotechnology-oriented drug delivery strategies whereby membrane composition of optical and nonoptical nanomedicines alike can be manipulated to tune pharmacokinetics. However, the photochemical and phototherapeutic consequences of liposomal membrane composition are significantly more complex. The presence of a membrane-inserting BPD-PC appears to reduce the rate of phototriggered calcein release by 12-fold and passive calcein release by 13-fold. Studies have previously explored the biochemical mechanisms of phototriggered agent release. While some studies have focused on the role of oxidizable unsaturated membrane phospholipids in facilitating phototriggered drug release [36], others have explored the role of PS degradation products in triggering membrane permeability upon photoexcitation [37]. Future studies into the photodegradation products of the membrane-inserting BPD-PC and the membrane-protruding DSPE-PEG-IRDye 700DX, their reactivities with membrane constituents, and their various phototoxicity profiles are therefore also warranted. With regards to phototoxicity, the radical-based photodegradation products of the individual PS-lipid conjugates are likely to provide insights into why the membrane-inserting lipo-BPD-PC exhibits such a marked increase in phototoxicity even though it is less efficient at generating RMS. This is also intriguing considering that no differences in cellular uptake efficiencies or subcellular localization exist between the two liposomal membrane compositions. It does, however, highlight the significance of the differences in the chemical nature of the two PS-lipid conjugates that give rise to the distinct conformations.

Overall, the findings of this study provide vital insights that can guide the application-specific and rational design and engineering of NIR-activable liposomes, whereby emerging combination therapies integrated into such platforms can be tailored to best suit the needs of the specific therapeutic modalities at hand. As such, the powerful attributes of NIR-activable liposomes and other lipid-based PNMs can be translated to maximal therapeutic benefits to patients with head and neck cancer, as well as patients with other cancer and noncancer indications whereby phototriggered combination therapy can prove to be pivotal. Furthermore, the rational combination of both compositions into the same construct can be leveraged to capitalize on the powerful attributes of the respective compositions.

## 4 Methods

### 4.1 Synthesis of DSPE-PEG-IRDye700DX and BPD-PC lipid conjugates

DSPE-PEG-IRDye700DX was synthesized using an adaptation of our previously published protocols [21, 38]. Briefly, 0.5 mg IRDye700DX-NHS (LI-COR) was dissolved in 100 μl anhydrous DMSO to make a 5 mg/ml solution. The IRDye700DX-NHS solution was added to dry DSPE-PEG_2000_-NH_2_ (Avanti) at a molar ratio of 1:1. This solution was stirred for 48 h at 2500 rotations per minute (RPM) at room temperature in the dark. The DSPE-PEG-IRDye700DX was purified by diluting the crude reaction mixture with 900 μl methanol and running through a Sephadex LH20 column (Cytivia) pre-equilibrated with methanol. Purified DSPE-PEG-IRDye700DX was stored at −20 °C in the dark in methanol.

The 20:0 lyso PC-benzoporphyrin derivative conjugate (BPD-PC) was synthesized as we previously described [18–21, 39]. Briefly, 1-arachidoyl-2-hydroxy-sn-glycero-3-phosphocholine (20:0 Lyso PC, Avanti), BPD (US Pharmacopeia), EDC (Sigma), 4-(Dimethylamino) pyridine (DMAP) (Sigma), and *N,N*-Diisopropylethylamine (DIPEA) (Sigma) were mixed at molar ratios of 1:5:50:25:60, respectively, in 5 ml of dichloromethane (Fischer Scientific, high-performance liquid chromatography [HPLC] grade) and stirred at 2500 RPM for 72 h at room temperature. BPD-PC was purified using preparatory thin-layer chromatography and extracted in a 2 : 1 dichloromethane/methanol mixture. The extracted BPD-PC was finally filtered through a 0.22 μm polytetrafluoroethylene (PTFE) filter and stored at −20 °C in the dark in chloroform.

### 4.2 Synthesis of liposomes

For the synthesis of lipo-IRDye700DX, 1,2-dipalmitoyl-*sn*-glycero-3-phosphocholine (DPPC) (Avanti), cholesterol (Avanti), DSPE-mPEG_2000_ (Avanti), and DSPE-PEG-IRDye700DX were mixed in chloroform at a molar ratio of 0.67, 0.30, 0.025, and 0.005, respectively. For the synthesis of lipo-BPD-PC, the lipids DPPC, cholesterol, DSPE-mPEG_2000_, and 20:0 lyso-PC-BPD were mixed in chloroform at a molar ratio of 0.665, 0.30, 0.03, and 0.005, respectively. Liposomes were prepared using a conventional thin-film hydration-extrusion method, by hydration in PBS and extrusion through 100 nm polycarbonate membranes (Whatman) [18–21, 39].

### 4.3 Characterization of liposomes

The hydrodynamic diameters, polydispersity indices, and *ζ*-potentials of the lipo-IRDye700DX and lipo-BPD-PC were performed by dynamic light scattering using the Zetasizer Pro (Malvern). 2 μL of each liposome were mixed with 1 mL of PBS, and the hydrodynamic diameters and polydispersity indices were measured in triplicate. *ζ*-potentials were measured in triplicate using 5 μL of each liposome mixed with 1 mL of 0.9% NaCl solution in MiliQ H_2_O.

To calculate the concentrations of DSPE-PEG-IRDye700DX and BPD-PC equivalents in the respective formulations, lipo-IRDye700DX and lipo-BPD-PC were diluted in DMSO and the absorbance was measured (Thermo Evolution 350 Spectrophotometer), using the molar extinction coefficient, *ε*_687_ = 34,895 M^−1^ cm^−1^ for BPD-PC [21] and *ε*_687_ = 210,000 M^−1^ cm^−1^ (LI-COR) for DSPE-PEG-IRDye700DX. Additional absorption and emission spectra were measured in a Tecan Spark plate reader.

### 4.4 Quantification of photogenerated RMS

Singlet oxygen was measured using the colorimetric probe anthracene-9,10-dipropionic acid (ADPA; Fisher Scientific) and with the fluorogenic probe Singlet Oxygen Sensor Green (SOSG; Fisher Scientific). For the ADPA singlet oxygen measurement assay, samples were prepared at 5 μM PS equivalent concentrations either in PBS (for liposomes, free IRDye700DX, and Cet-IRDye700DX) or in DMSO (for DSPE-PEG-IRDye700DX and BPD-PC). The samples in PBS were placed in 96 well plates in 100 μL aliquots, and 5 μL of ADPA (6 mM stock in methanol) was added to each plate. The same was repeated for the samples in DMSO, although 10 μL of ADPA (6 mM stock in methanol) was used to account for the lower absorbance of ADPA in DMSO. The absorbance of all samples containing ADPA was measured from 200 to 800 nm using a Tecan Spark Plate reader before and after irradiation with 690 nm LED light. Samples were irradiated using an irradiance of 17.86 mW cm^−2^ up to a total fluence of 3 J cm^−2^, with absorbance spectra being measured in 0.5 J cm^−2^ increments.

For the SOSG singlet oxygen measurement assay, samples were prepared at 5 μM PS equivalent concentrations in PBS and placed in 96 well plates in 100 μL aliquots. 10 μL of SOSG (50 μM stock) were added to each sample and fluorescence emission was measured from 500 to 600 nm using an excitation wavelength of 460 nm on the Tecan Spark Plate reader. Samples were irradiated with 690 nm LED light using an irradiance of 17.86 mW cm^−2^ up to a total fluence of 3 J cm^−2^, with fluorescence spectra being measured in 0.5 J cm^−2^ increments.

Hydroxyl radicals and peroxynitrite radicals were measured using the fluorogenic probe hydroxyphenyl fluorescein (HPF, Fisher Scientific). Samples were prepared at 5 μM PS equivalent concentrations and placed in 96 well plates in 100 μL aliquots. 20 μL of HPF (200 μM stock) were added to each sample and fluorescence emission was measured from 500 to 600 nm using an excitation wavelength of 460 nm on the Tecan Spark Plate reader. Samples were irradiated with 690 nm LED light using an irradiance of 17.86 mW cm^−2^ up to a total fluence of 3 J cm^−2^, with fluorescence spectra being measured in 0.5 J cm^−2^ increments.

### 4.5 Passive and phototriggered calcein release

Lipo-IRDye700DX and lipo-BPD-PC were both prepared as described earlier and hydrated with 100 mM calcein disodium salt (Sigma) dissolved in PBS as an optical surrogate for water-soluble drug payloads. Calcein-loaded liposomes were purified from unentrapped calcein using size exclusion chromatography by running on a Sepharose CL-48 column (Sigma) equilibrated with PBS. To calculate the concentration of calcein in each liposomal formulation, lipo-IRDye700DX and lipo-BPD-PC were diluted in DMSO, the absorbance was measured (Thermo Evolution 350 spectrophotometer), and the concentration was calculated using the molar extinction coefficient, *ε*_495_ = 80,000 M^−1^ cm^−1^ [40]. To measure passive and phototriggered release, samples of each liposome were prepared at concentrations of 5 μM of calcein equivalent of purified lipo-IRDye700DX and lipo-BPD-PC. The fluorescence emission of the liposomes was measured from 500 to 600 nm with an excitation wavelength of 460 nm using a Tecan Spark Plate reader. For the passive release studies, liposomes were incubated in a 37 °C incubator, and the fluorescence dequenching of calcein as it escaped the liposomes was measured at 1, 3, 6, and 24 h of incubation. For the phototriggered release studies, the liposomes were irradiated after purification at room temperature by a 690 nm LED system (BioLambda; 17.86 mW cm^−2^) in 10 J cm^−2^ increments up to 50 J cm^−2^. The fluorescence dequenching of calcein as it escaped the liposomes was measured following each irradiation fraction.

### 4.6 Cellular uptake of liposomes and PS variants

FaDu cells were seeded in 96-well plates at a density of 50,000 cells per well in Dulbecco’s Modified Eagle Medium (DMEM). After 24 h, 250 Nm PS equivalent of lipo-IRDye700DX and lipo-BPD-PC, as well as free IRDye700DX, free BPD at Cet-IRDye700DX were added to cells and further incubated for another 24 h. Media was then aspirated, and cells were washed with 200 μl of PBS three times. After washing, 100 μl of DMSO was added to each well and the fluorescence emission was measured at 670–800 nm, using an excitation wavelength of 630 nm using a Tecan Spark Plate reader. Standard curves of each PS in their respective formats (i.e. free, liposomes, conjugates, etc.) were prepared in DMSO and used to calculate PS concentrations taken up by cells.

### 4.7 Confocal microscopy

FaDu cells were seeded in glass-bottom 96-well plates at a density of 50,000 cells per well. After a 48 h incubation at 37 °C, lipo-IRDye700DX and lipo-BPD-PC were added to the cells at a 2000 nM PS equivalent and were further incubated for 24 h. Prior to imaging, the liposomes were removed from the cells and fresh media was added containing either Hoechst 33342 or LysoTracker^™^ Green DND-26, as per the instructions of ThermoFisher Scientific. The Hoechst and LysoTracker^™^ were incubated with the cells for 30 min and the cells were imaged using a Leica SP8 microscope with a 405 nm laser (Hoechst excitation), a 488 nm laser (LysoTracker^™^ excitation), a 647 nm laser (lipo-IRDye700DX and lipo-BPD-PC excitation), and a 60× oil immersion objective.

## 5 Photodynamic therapy

FaDu cells were seeded in transparent 96-well plates at a density of 1500 cells per well. After 24 h incubation at 37 °C, lipo-IRDye700DX and lipo-BPD-PC, as well as free IRDye700DX, free BPD at Cet-IRDye700DX were incubated with the cells for a further 24 h at 5, 50, 500, and 2000 nM PS equivalent. After incubation, cells were irradiated using the 690 nm BioLambda LED system with various fluences: 0, 1, 2.5, 5, and 10 J cm^−2^ at a consistent irradiance of 17.86 mW cm^−2^. After irradiation, cells were incubated for 48 h then assessed using the 3-(4,5-Dimethylthiazol-2-yl)-2,5-diphenyltetrazolium bromide (MTT, Sigma) assay. The absorbance of the MTT product formazan was measured at 555 nm using a Tecan Spark Plate reader.

### 5.1 Biodistribution and tumor imaging in orthotopic head and neck tumors

7.5 × 10^5^ FaDu cells in sterile PBS were implanted transcervically into the floor of the mouth of male Swiss Nude Mice (20 g, 4–6 weeks old, Charles River) [41]. Tumor development was monitored using ultrasound imaging (Vevo 3100 VisualSonics). Once tumors reached 5 mm in diameter, mice were intravenously injected with 6.95 nmol PS equivalent of lipo-IRDye700DX and lipo-BPD-PC. Longitudinal fluorescence imaging of the liposomes in the tumors was performed using an LI-COR PEARL system. Tumors were also imaged using fluorescence tomography/micro-computed tomography (FLT/μCT, MI Labs). At 24 h after administration, tumors and organs were harvested, liposome content was quantified using the LI-COR PEARL system and tumor penetration was imaged in bisected tumors using the LI-COR Odyssey system. All images were corrected for autofluorescence background signals and for the inherent variability in fluorescence intensities of the two constructs in the respective imaging systems. Tumor accumulation was quantified using the LI-COR PEARL software and tumor penetration was quantified using the 3D surface plot tool on ImageJ (NIH).

## Supplementary Material

SI

## Figures and Tables

**Table 1:** Fluorescence emission properties of each PS in its native form, when conjugated to its respective lipid, and when formulated into liposomes.

Sample	Solvent	Emission wavelength maxima (nm)	% Change in fluorescence emission
IRDye700DX	DMSO	692	N/A
DSPE-PEG-IRDye700DX	DMSO	698	+15.1% (upon lipid conjugation)
lipo-IRDye700DX	PBS	700	−25.0% (upon formulation)
BPD	DMSO	698	N/A
BPD-PC	DMSO	698	0% (upon lipid conjugation)
lipo-BPD-PC	PBS	698	−74.2% (upon formulation)

**Table 2:** ζ-potentials and hydrodynamic diameters of lipo-IRDye700DX and lipo-BPD-PC in PBS.

	lipo-IRDye700DX	lipo-BPD-PC
ζ-Potential (mV)	−7.67 ± 1.65	−2.09 ± 0.47
Hydrodynamic diameter (nm)	144.4 ± 0.68	138.1 ± 0.99

(Values are mean ± standard deviation).
