# Supplementary material for: Membrane composition is a functional determinant of NIR-activable liposomes in orthotopic head and neck cancer
Source: Nanophotonics. Author manuscript; Available in PMC 2022 Apr 15. (PMC9012185; doi:10.1515/nanoph-2021-0191)
Supplement: SI [file NIHMS1774682-supplement-SI.pdf]

SUPPORTING INFORMATION

Membrane composition is a functional determinant of NIR activable liposomes in orthotopic head and neck cancer

Mina Guirguis[a†], Chanda Bhandari[a†], Junjie Li[a], Menitte Eroy,[a] Sushant Prajapati[a], Ryan Margolis[a], Navadeep Shrivastava[a], Kenneth Hoyt[a], Tayyaba Hasan[b,c] and Girgis Obaid[a*]

[a] Department of Bioengineering, University of Texas at Dallas, Richardson, Texas 75080, U.S.

[b] Wellman Center for Photomedicine, Massachusetts General Hospital and Harvard Medical School, Boston, Massachusetts 02114, U.S.

[c] Division of Health Sciences and Technology, Harvard University and Massachusetts Institute of Technology, Cambridge, Massachusetts 02139, U.S.

† Both authors contributed equally to this study

*Corresponding author email: Girgis.Obaid@utdallas.edu

Supplementary Results

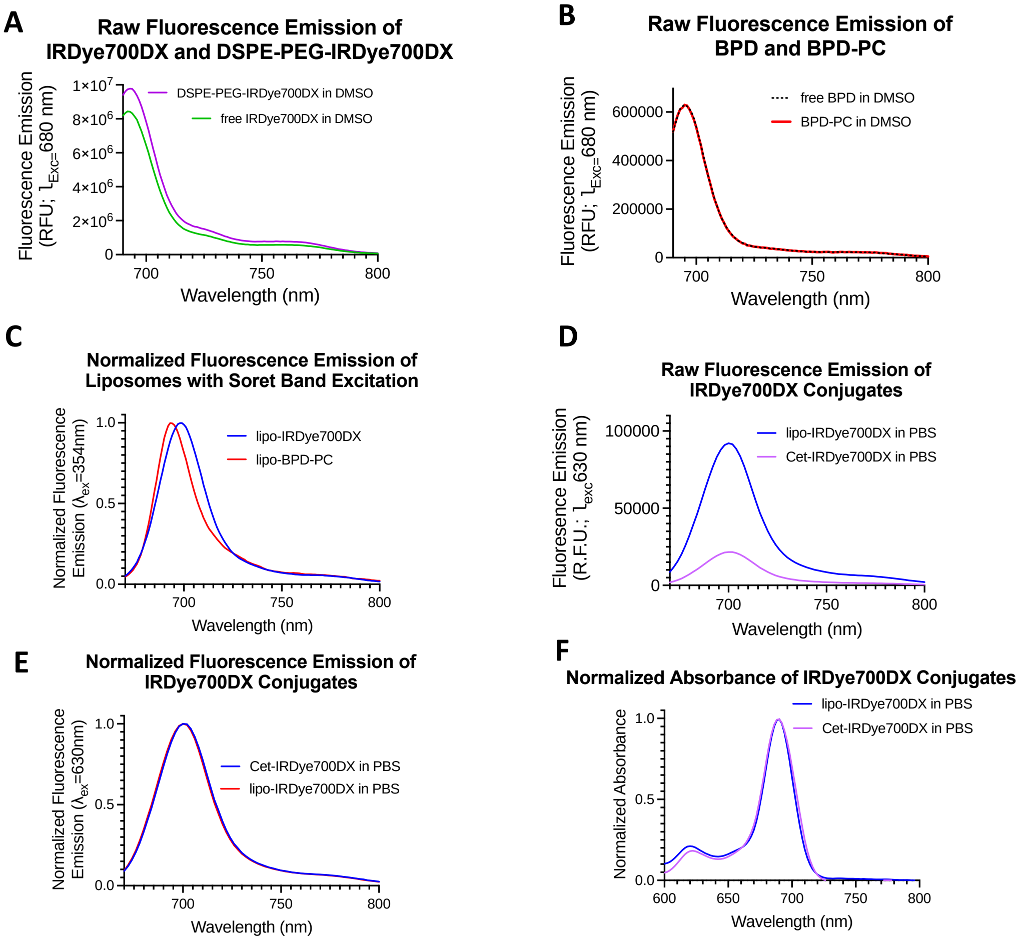

Figure S1. Raw fluorescence emission of IRDye700DX and DSPE-PEG-IRDye700DX in DMSO (A), and BPD and BPD-PC in DMSO (B). C) Normalized fluorescence emission spectra of lipo-IRDye700DX and lipo-BPD-PC upon excitation of the Soret bands. Raw fluorescence emission (D) and normalized fluorescence emission spectra (E) of lipo-IRDye700DX and Cet-IRDye700DX. Normalized absorbance spectra of the Q-band of lipo-IRDye700DX and Cet-IRDye700DX.

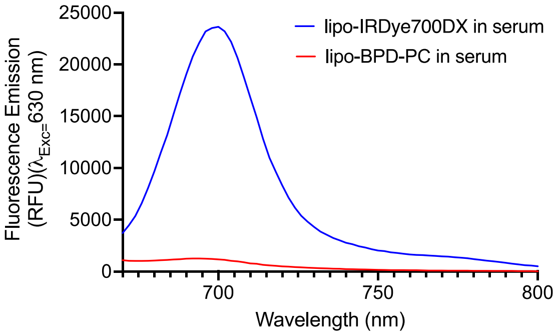

Figure S2. Raw fluorescence emission spectra of lipo-IRDye700DX and lipo-BPD-PC in pure serum (bovine) upon excitation at 630 nm that simulates the fluorescence imaging parameters on the µCT/FLI system.

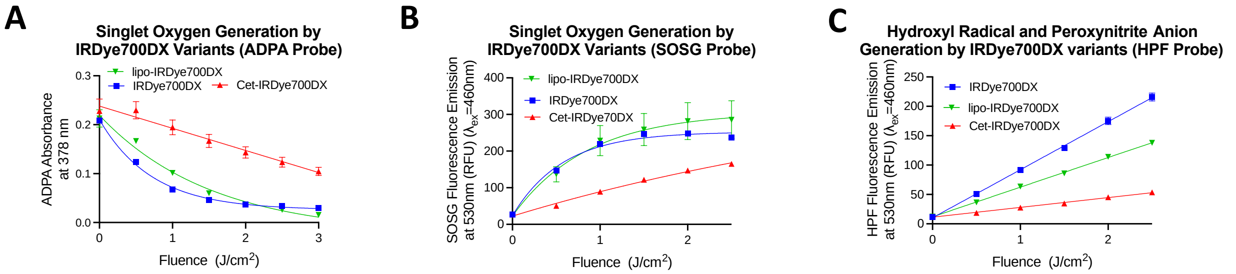

Figure S3. Generation of reactive molecular species by free IRDye700DX, lipo-IRDye700DX and Cet-IRDye700DX using the singlet oxygen probe ADPA (A), the singlet oxygen probe Singlet Oxygen Sensor Green (B) and hydroxyl radical and peroxynitrite probe HPF (C).

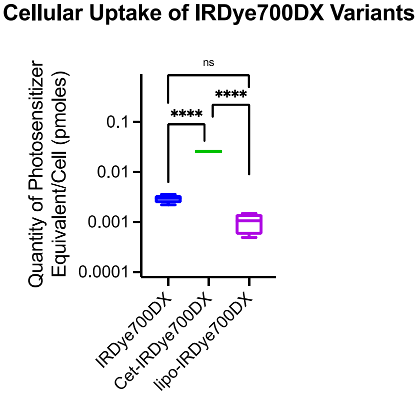

Figure S4: Comparison of FaDu cellular uptake of IRDye700DX variants: lipo-IRDye700DX, free IRDye700DX and Cetuximab-IRDye700DX conjugates after 24 h incubation. (Statistical significance was calculated using one-way ANOVA with a Tukey post-test, ****: $P \leq 0.0001$).

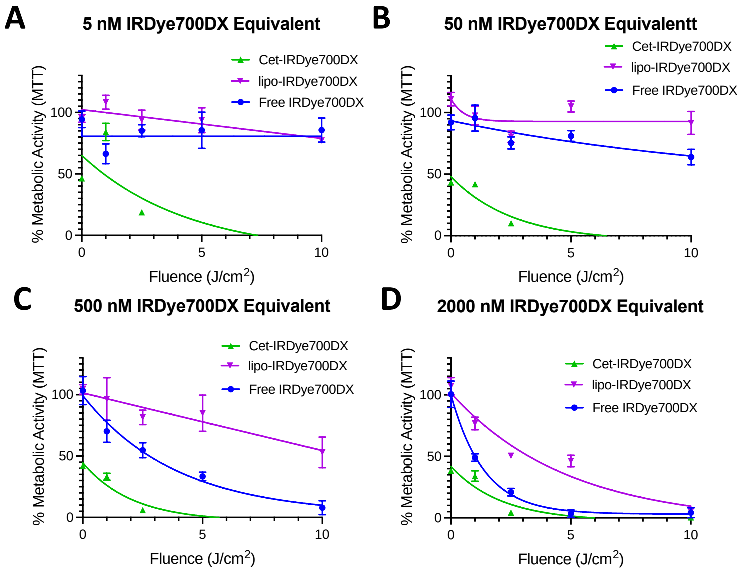

Figure S5: Comparison of FaDu cell phototoxicity with IRDye700DX variants: lipo-IRDye700DX, free IRDye700DX and Cetuximab-IRDye700DX conjugates after 24 h incubation. Concentrations were at 5 nM (A), 50 nM (B), 500 nM (C), and 2000 nM (D) photosensitizer equivalent and irradiation at 690 nm light was used with an irradiance of 27.7 mW.cm^{-2}. (Data is mean ± standard error).
